# Acculturation and mental health among adult forced migrants: a meta-narrative systematic review protocol

**DOI:** 10.1186/s13643-019-1103-8

**Published:** 2019-07-25

**Authors:** Brittney S. Mengistu, Gergana Manolova

**Affiliations:** 1 Department of Global Health & Social Medicine, King’s College London, Bush House NE Wing, 30 Aldwych, London, WC2B 4BG UK; 2 Global Public Health, Migration & Ethics Research Group, Faculty of Medical Science, Anglia Ruskin University, Chelmsford, UK; 3 Institute of Psychiatry, Psychology and Neuroscience, King’s College London, London, UK

**Keywords:** Acculturation, Adult, Biculturalism, Forced migrants, Mental Health, Meta-narrative review

## Abstract

**Background:**

The global refugee crisis has become central to health and policy debates. There is a growing need to investigate how acculturation impacts mental health among asylum seekers and refugees. Many forced migrants have an increased risk of experiencing negative mental health outcomes, but this review will only assess the current literature on acculturation and mental health among adults. Research questions include the following: (1) How is acculturation conceptualised? (2) What are the most salient mental health outcomes? (3) How are acculturation and mental health measured and related? and (4) How do macrostructural factors affect the relationship between mental health and acculturation?

**Methods:**

We will use a meta-narrative approach to synthesise the breadth of acculturation and mental health literature from various research traditions. This review will include empirical studies measuring variations of acculturation and mental health among adult forced migrants from low- and middle-income countries residing in high-income countries. Studies will be retrieved from the following academic databases: MEDLINE, Embase, PsycINFO, Global Health, ProQuest Social Science and Web of Science. Additional studies will be collected from King’s College London’s online library databases and e-resources, and reference lists of eligible studies. Studies from database inception and written in English will be included. All full-text papers will undergo quality appraisal using the Mixed Methods Appraisal Tool (MMAT). Extracted data will be analysed using a conceptual framework analysis to construct overarching narratives and a framework that will describe the relationship between acculturation and mental health.

**Discussion:**

A meta-narrative systematic review provides a flexible and systematic approach to synthesising the heterogeneous literature on acculturation and mental health. This review will guide the development of a conceptual framework to aid future research on acculturation among adult forced migrants. As high-income countries seek to integrate forced migrants into society and improve their access to vital resources, this review has the potential to transform policies and practices that influence migrant mental health.

**Systematic review registration:**

PROSPERO CRD42018089148

**Electronic supplementary material:**

The online version of this article (10.1186/s13643-019-1103-8) contains supplementary material, which is available to authorized users.

## Background

By the end of 2016, the total number of forcibly displaced persons increased by 300,000 people totalling over 65 million displaced persons around the world [1]. Millions of forced migrants have taken refuge in neighbouring countries, but many continue their journey to European and North American countries [1]. Many experience symptoms of mental illness upon arrival to their host country due to traumatic pre-emigration and migration experiences [2–5]. Socio-demographics and post-migration environment can negatively affect their mental health outcomes [6, 7] and have the potential to exacerbate existing mental health symptoms. The acculturation process, broadly defined as the means of balancing aspects of the receiving and heritage country’s culture, can be stressful to a newly resettled migrant [8–10].

Acculturation was initially assumed to be unidirectional, where individual identity is located on a continuum ranging from the heritage country’s culture to the receiving country’s culture [11]. However, advancements in acculturation psychology have challenged a unidimensional perspective because it excludes individuals who identify with multiple cultures or those who do not actively identify with any cultural group [12]. This concern has prompted scholars to further investigate the complexities of cultural identity, values and behaviour. This exploration resulted in the emergence of bi- and tridimensional paradigms and a multidimensional approach to acculturation conceptualisation [13, 14]. A bidimensional model posits that individuals can fully adopt aspects of one culture while maintaining aspects of their own culture [9] and a tridimensional paradigm proposes that individuals can be entirely oriented towards three cultures [15]. Further, Schwartz et al. [14] argue that the acculturation process not only is limited to identity, but also includes a reciprocal exchange of cultural values and practices between the receiving and host country.

Acculturation conceptualisation has evolved, and its operationalisation continues to vary across different research domains. Many studies in psychology, sociology, public health and anthropology have referred to acculturation as *integration*, *assimilation* or *adaptation*, but these terms fail to describe the complexity of individual and group-level cultural change. Conceptualisations and perceptions about the acculturation process affect how it is measured and its effect on hypothesised outcomes [16], such as mental health.

Literature has shown that acculturating immigrants or ethnic minorities not only have an increased risk of substance use [17] and poor mental health outcomes [18], but also display positive help-seeking attitudes and behaviours [19, 20]. Parental and adolescent relationships [21] and pre- and post-natal experiences [22–24] are shown to affect the acculturation process and mental health outcomes negatively. Forced migrants may share similar experiences with acculturating immigrants [25], as many feel uncertain about integrating into a new society and adopting aspects of a new culture [26].

Additional factors that may influence the relationship between acculturation and mental health can be macrostructural, such as the host country’s socio-political climate, migrants residing in multicultural cities and other societal influences that are beyond the migrant’s control. For example, direct exposure to newly arrived asylum seekers increased Greek natives’ hostility towards refugees, immigrants and Muslims [27]. This growing hostility can be seen amidst the ‘EU Refugee Crisis’, as the political right in the EU is in favour of exclusionary policies and stricter migration control than the political left [27, 28]. Socially, many migrants may be exposed to competing host cultures within multilingual, multicultural cities or regions across the EU, thus challenging the bidimensionality of the acculturation process [29]. Phalet and Kosic [29] also describe how prejudices and exclusionary practices from the host society, such as employment discrimination and exclusion from communities and neighbourhoods, affect the migrant’s ability to integrate into the host society. These contextual factors, though they may not be explicitly measured in this field of research, highlight important considerations when assessing migrants’ experiences with acculturation.

Previous systematic reviews and meta-analyses have explored acculturation and mental health among Hispanic immigrants [30] and racial and ethnic minorities [31–33]. These outcomes may be similar for forced migrants; however, the concept of involuntary acculturation is not captured within these studies. Moreover, the relationship between acculturation and mental health among forced migrants has not been described in the literature. Synthesising the research on acculturation and mental health is undoubtedly important in understanding the experiences of forced migrants, but these experiences differ between adults and adolescents. Many youths interact with the receiving country’s culture through school and social activities, and mental illnesses can impede their academic functioning [34]. We feel that including youths in this review will minimise their unique experiences with identity formation and peer interactions, so this review will only include adult populations. Investigating the mental health effects of involuntary acculturation will provide a deeper understanding of the acculturation process and how macrostructural factors affect the relationship between these variables.

## Research objectives

The overall aim of this review is to systematically assess the literature on acculturation and mental health among adult forced migrants and create a conceptual framework describing the relationship between acculturation and mental health. The framework will not be a one-size-fits-all but a guiding model for policy, practice and future research.

This review seeks to answer the following questions:

1. How is acculturation conceptualised?
2. What are the most salient mental health outcomes?
3. How are acculturation and mental health measured and related?
4. How do macrostructural factors affect the relationship between mental health and acculturation?

This review will only consider studies which have collected primary or empirical data, regardless of comparator groups. Study participants will be asylum seekers or refugees from low- and middle-income countries who are experiencing acculturation, biculturalism or cultural adaptation in high-income countries. Income classifications will be sourced directly from the World Bank, which will provide the most current classifications of each country’s income. The outcome of interest includes self-reported or perceived mental health outcomes, experiences with mental health help-seeking, which includes behaviours, attitudes, intentions and health service use.

## Methods and design

Several research traditions, such as neuroscience, anthropology, public health, psychology and psychiatry, have investigated acculturation and mental health. Discipline-specific training has resulted in scholars adopting varied theoretical, conceptual, methodological and instrumental approaches to similar research questions [35]. An anthropologist may envisage acculturation to be a fluid process that is part of the resettlement experience and qualitatively explore the mental health effects of settlement. Conversely, a psychiatrist may describe acculturation to be a bi-dimensional construct that is quantifiable and statistically correlated with mental health outcomes, such as post-traumatic stress disorder, depression or anxiety. The different approaches to acculturation and mental health inquiry pose several challenges in conventional systematic review data synthesis.

A narrative synthesis is a traditional approach to reporting qualitative and mixed methods systematic reviews. This approach textually summarises quantitative and qualitative data, identifies and explores the relationship between emerging themes and assesses the robustness of the review findings [36]. Constructing narratives requires reviewers to consider the contextual factors of articles in the review, but a meta-narrative approach further analyses the narratives to explore how the phenomenon has changed over time. Documenting how this research agenda has evolved is an essential component of this review, as capturing the research progression in various academic traditions will delineate how methodologies have evolved. The evolution of this research is especially relevant and timely due to the dramatic increase in mass migration and refugee mental health research.

A meta-narrative systematic review synthesises the breadth of literature from various academic domains, critically assesses how the study contributes to the literature and constructs overarching meta-narratives that informs policy, practice and future research [37]. This approach highlights how research traditions evolved, the differences in their approach and the similar and contrasting methods and outcomes [35]. A meta-narrative approach is most suitable for this review because it provides a pragmatic and descriptive approach to literature synthesis that recognises and highlights the diversity of the various research traditions.

A meta-narrative systematic review consists of six phases: planning, searching, mapping, appraising, synthesising and providing recommendations [35]. The planning phase includes seeking collaborators to assist with refining the review questions and conducting the systematic review. The searching phase involves creating a search strategy and systematically identifying eligible studies. The mapping phase requires identifying characteristics of various research traditions and academic domains of the literature being assessed. The appraisal phase includes critically evaluating each article for its relevance and inclusion in the review. The synthesis phase consists of synthesising the relevant literature, constructing overarching narratives and highlighting contradictory findings. Lastly, the recommendation phase will include policy, practice and research recommendations.

**Fig. 1:**
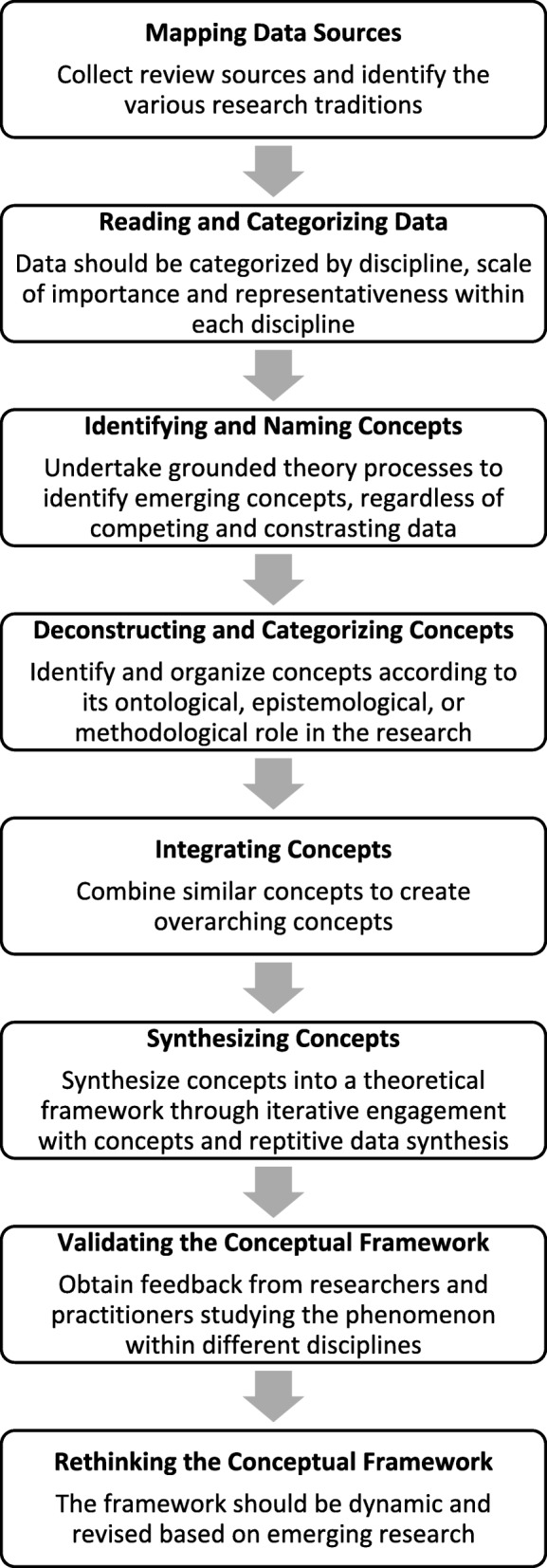
Conceptual framework analysis

A meta-narrative approach is sufficient in reporting findings to a broad audience, but additional analysis is warranted to generate theoretical concepts for framework development. Further analysis will be undertaken to create a conceptual framework that illuminates how dimensions of acculturation and mental health may interact. Conceptual framework analysis (CFA) is an iterative, theoretical approach to generating concepts from heterogeneous data and synthesising ideas to create a conceptual framework [38]. This analysis occurs in eight phases: mapping data sources, reading and categorising data, identifying and naming concepts, deconstructing and categorising concepts, integrating concepts, synthesising the data, validating the conceptual framework and rethinking the conceptual framework (Fig. 1). CFA will be used during the meta-narrative synthesis phase.

The meta-narrative systematic review training materials [37] and publication standards [39] were used to design this systematic review protocol. The Preferred Reporting Items for Systematic Review and Meta-Analysis Protocols (PRISMA-P) was also used to guide the development of this protocol (Additional file 1) [40]. This protocol is registered with PROSPERO, the international prospective register of systematic reviews (CRD42018089148).

### Planning phase

A multidisciplinary team was assembled to undertake this systematic review. BSM conceptualised the systematic review and drafted the original review questions and systematic review protocol. GM assisted in refining and finalising the systematic review protocol. Two external researchers provided feedback on the systematic review protocol and will be consulted throughout the review if additional assistance is required.

### Searching phase

Empirical studies will be accessed from the following academic databases: MEDLINE, Embase, PsycINFO, Global Health, ProQuest Social Science and Web of Science. Additional peer-reviewed articles will be accessed through King’s College London online library database, which provides access to over 500 academic databases and e-journals. This exhaustive database will provide access to relevant articles that are not included in the academic databases previously listed. Articles published from database inception up until February 2018 and written in English are eligible for inclusion. Reference lists of all eligible studies will be searched to identify articles that were undetected by electronic searches.

The search strategy will include a combination of Boolean operators and MeSH and search terms that will be formatted for each database (Additional file 2). Qualitative, quantitative and mixed methods studies will be eligible for the review if they meet the following criteria:

1. Measure acculturation, biculturalism or cultural adaptation,
2. Measure mental health outcomes, perceived mental health, experiences with seeking mental health care and experiences with mental health help-seeking, which includes behaviours, attitudes, intentions and/or health service use,
3. Participants must be adult refugees or asylum seekers, aged 18 and older, from low- or middle-income countries.
4. Research must be done in a high-income country.
5. The study must be written in English.
6. The study must present primary, empirical data.

Studies on adolescent refugees with participants older than 18 will be further assessed for inclusion. If studies report aggregate data including adolescent and adult refugees, then the findings will be summarised separately from the adult-only results. All studies meeting the above criteria will be included in the review, regardless of a control group.

All records will be imported and managed using the EndNote citation manager. Multiple EndNote folders will be created to track the number of records throughout each stage of the review process. Duplicate records will be deleted before the initial screening phase. The screening process will occur in two rounds: title and abstract, and full-text. Both reviewers will assess a sample of the records at the beginning of each screening round to establish screening consistency and inter-rater agreement. After reaching at least 80% agreement, the reviewers will independently screen the remaining articles. If a reviewer is unsure about the study’s relevance to the systematic review, the two reviewers will meet and reach an agreement on its inclusion during each round of the screening process. If the two reviewers are unable to reach a consensus, additional reviewers will be recruited and consulted. Reasons for elimination will be provided for each record during both stages of the screening process. The number of records and reasons for exclusion during each round will be recorded in the PRISMA diagram [40].

### Mapping phase

A thorough data extraction spreadsheet will be piloted on a sample of eligible studies to ensure that all relevant data is captured. The data extraction spreadsheet adapted from Noyes [41] will record the research tradition, study setting, research questions, theoretical background, participant characteristics, data collection and analysis methods, results, methodological quality and additional information specific to review questions. After reviewers reach consensus on the utility of the data extraction spreadsheet, it will be refined and used to extract data from remaining records. Two reviewers will then independently extract data from the remaining eligible studies. At the end of data extraction, a table will be created summarising the main findings of each study.

### Appraisal phase

All studies will be assessed for quality using the Mixed Methods Appraisal Tool (MMAT) after evaluating full-text records for eligibility. This tool contains 21 items covering five methodological domains: qualitative, quantitative randomised control trials, quantitative non-randomised, quantitative descriptive and mixed methods [42]. MMAT is an efficient screening tool and commonly used to assess methodological quality for systematic review articles, and it is advisable that reviewers meet to establish a mutual understanding of MMAT’s assessment criteria to ensure consistency [43]. All reviewers will meet to discuss the MMAT’s usability and assess a sample of full-text articles. Reviewers will independently evaluate the remaining full-text articles after reaching more than 80% agreement on quality assessment. The results of each study will be assessed together with its methodological quality. If the quality of the overall paper is low, the article may be excluded from analysis as long as all reviewers have agreed to its exclusion.

### Synthesis phase

**Fig. 2:**
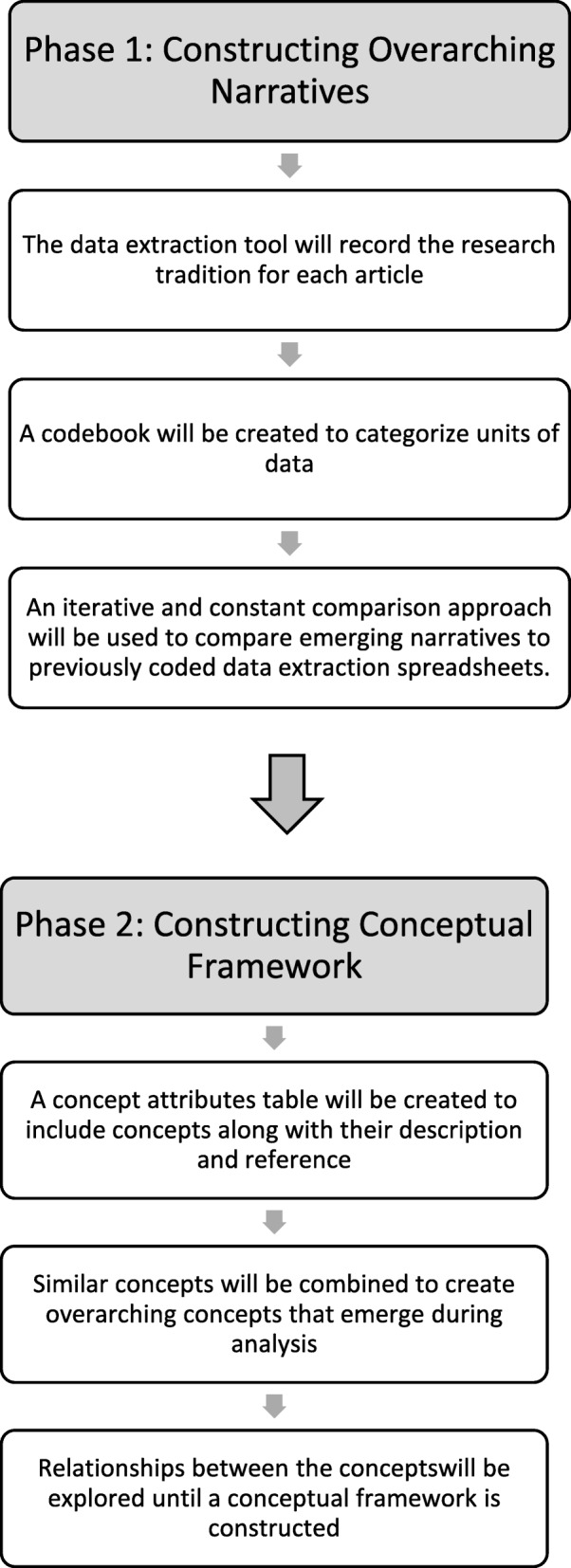
Data analysis phases

The synthesis of findings will occur in two phases: constructing overarching narratives and building a conceptual framework (Fig. 2). This approach is influenced by grounded theory, whereby reviewers take an iterative approach to data analysis and extraction [38]. Like grounded theory, reviewers will use open (data categorisation), axial (identifying relationships) and selective (describing phenomenon) coding to identify units of meaning within the data [44, 45]. The analysis will commence during data extraction to apply new and emerging codes and categories to subsequent data. Through abductive analysis, reviewers will create inferences about the data by identifying emerging themes and observing the relationship between them [46]. Reviewers will use a constant comparison approach [47] to contrast emerging review findings to previous data extraction sheets, which will provide a systematic approach to identifying narratives and concepts.

We will create a preliminary codebook with deductive codes informed by the data extraction sheet and inductive codes that emerge during data analysis. Codebook revision and data analysis will occur iteratively, as the codebook will be revised to capture and describe the data. Analysis and codebook revisions will occur iteratively until an exhaustive list of codes has been created. The data extraction sheets and codebook will be imported into NVivo 11 for data management and analysis. Upon completion of data analysis, reviewers will meet to discuss emerging themes and overarching narratives. We will then identify the narratives of each research tradition and explore how these narratives have unfolded over time. These narratives will be explored further to understand the theoretical, conceptual, methodological and instrumental approaches in each research tradition. Due to the varied epistemological differences between and within the social science disciplines, we anticipate diverse conceptual approaches to understanding mental health outcomes of forced migrants. As a result, we will compare the similarities and differences between these narratives to create meta-narratives that summarise conceptual, theoretical and methodological praxis and critical debates in the literature [39]. These meta-narratives will highlight tensions and contradictory findings within the literature [35].

We will begin the CFA by re-examining the data to identify and deconstruct emerging concepts. This process includes identifying ontological, epistemological and methodological contributions to the literature [38]. Concepts will be synthesised to create a conceptual framework that is flexible and amenable to change. Berry [16] urges investigators to refrain from creating generalisations about groups of individuals, such as ‘minorities’ and ‘refugees’, experiencing acculturation. Therefore, this conceptual framework will serve as a guide for future refugee mental health research.

The last components of CFA include validating and rethinking the conceptual framework. Validation from scholars studying the same or similar phenomenon can help refine the conceptual framework, which is similar to member checking in grounded theory analysis. Therefore, the systematic review will be submitted for publication in a high-impact, multidisciplinary journal where constructive criticism will be elicited from refugee mental health scholars within varying research domains. The publication of these findings will encourage further discussions within the acculturation, mental health and refugee research communities on the application and utility of the framework.

### Recommendations phase

The team will reconvene to discuss recommendations for future research, practice and policy after validating the findings. The overarching narratives will be primarily tailored for policymakers and mental health practitioners. Policy and practice recommendations will address practical ways to meet the mental health needs of growing refugee communities in high-income countries. The conceptual framework will be tailored to an academic audience because of its theoretical contributions to the literature. Research recommendations will explain how to advance the theoretical, conceptual, methodological and instrumental approaches to acculturation and refugee mental health research.

## Discussion

Refugee mental health has become increasingly important during an unprecedented international refugee crisis [7]. Many asylum seekers and refugees undergo acculturation involuntarily, which has the potential to negatively impact mental health [25]. Unfortunately, the literature on acculturation and mental health among forced migrants varies by research tradition and is complex and often inconsistent [25]. These experiences also differ by age, as youths and adults are presented with different social settings, and cultural maintenance and identity formation processes. This review will solely focus on adult forced migrants to capture their unique experiences. A meta-narrative approach will be used to systematically synthesise the breadth of literature from diverse academic domains and construct overarching narratives describing the current knowledge base on this phenomenon. Further, a conceptual framework will be created to theoretically represent the relationship between acculturation and mental health and to advance future acculturation and mental health research.

To our knowledge, this is the first systematic review to synthesise the literature on acculturation and mental health among adult forced migrants. A systematic search strategy will be used to identify and collect academic literature, and all eligible articles will be assessed for methodological quality. Data will be extracted and analysed using grounded theory. This analysis will identify emerging narratives and concepts that will be synthesised into overarching narratives and a conceptual framework. By engaging scholars and members of refugee communities with the results of this review, we hope that the narratives and framework will be further refined to provide practical recommendations for researchers, policymakers and mental health practitioners.

## Additional files

Additional file 1: Preferred Reporting Items for Systematic review and Meta-Analysis Protocols (PRISMA-P) 2015 checklist. (DOCX 32 kb)

Additional file 2: Search strategy to be used. (DOCX 15 kb)

## Data Availability

Not applicable.

## Abbreviations

BSM: Brittney S. Mengistu
CFA: Conceptual framework analysis
GM: Gergana Manolova
MeSH: Medical Subject Headings
MMAT: Mixed Methods Appraisal Tool
PRISMA: Preferred Reporting Items for Systematic Review and Meta-Analysis
PRISMA-P: Preferred Reporting Items for Systematic Review and Meta-Analysis Protocols
