# Supplementary material for: Acculturation and mental health among adult forced migrants: a meta-narrative systematic review protocol
Source: Syst Rev. 2019 Jul 25;8:184. doi: 10.1186/s13643-019-1103-8 (PMC6657160; doi:10.1186/s13643-019-1103-8)
Supplement: Supplementary file 2 — Search strategy to be used. (DOCX 15 kb) [file 13643_2019_1103_MOESM2_ESM.docx]

1. Adult*.tw.

2. exp Adult/

3. or/1-2

4. exp Adolescent/ not adults.sh.

5. 3 not 4

6. (acculturat* or bicultur* or multicultur* or ethnic identity or westerni?ation or re-sociali?ation or adaptation or cultur* adaptation or integration or assimilation).tw.

7. exp Acculturation/

8. or/6-7

9. (refugee* or asylum seek* or displaced person or forced migrants).ab.

10. exp Refugee/

11. or/9-10

12. ((mental* adj2 (health or ill or illness* or disorder* or hygiene)).tw.

13. ((help seeking adj2 (behavi* or attitude* or intention*)) or (health seeking adj2 (behavi* or attitude* or intention*))).tw.

14. (health service adj2 (uptake or utili?ation)).tw.

15. exp Mental Health/

16. or/12-15

17. Qualitative.pt. or (interview* or focus group* or ethnograph* or case study).ab.

18. Quantitative.pt. or (cross-section* or case study or survey or questionnaire).ab.

19. (Longitudinal* or regression* or association* or relationship* or observation*).ab.

20. (Empirical or data or correlation*).ab.

21. exp Research/

22. or/17-21

23. 5 and 8 and 11 and 16 and 22

24. limit 23 to english language

25. remove duplicates from 24
